# SAT1‐Induced Spermidine Depletion Potentiates Food Allergy

**DOI:** 10.1002/advs.77798

**Published:** 2026-09-27

**Authors:** Manman Liu, Beiyu Zhang, Junjuan Wang, Yue Jiang, Shiwen Han, Qianwei Wang, Lingyu Zeng, Jinfeng Wang, Huilian Che

**Affiliations:** ^1^ College of Food Science and Nutritional Engineering China Agricultural University Beijing People's Republic of China; ^2^ Key Laboratory of Food Nutrition and Health Evaluation Technology State Administration for Market Regulation Beijing People's Republic of China; ^3^ Institute of Agri‐food Processing and Nutrition Beijing Academy of Agriculture and Forestry Sciences Beijing People's Republic of China

**Keywords:** autophagy, food allergy, lncRNA, spermidine/spermine N^1^‐acetyltransferase, spermidine

## Abstract

Food allergies affect over 8% of children globally, with a 3.5‐fold increase in incidence in the past two decades. However, disease‐modifying therapies remain limited due to incomplete understanding of the underlying metabolic mechanisms. Thus, in this study, we aimed to demonstrate if spermidine/spermine N^1^‐acetyltransferase (SAT1)‐mediated spermidine depletion drives food allergy pathogenesis through a previously unrecognized metabolic‐immunological axis. Using a peanut allergy mouse model complemented by additional food allergen systems, we show that spermidine levels are significantly reduced during allergic responses, while exogenous supplementation attenuates disease severity. Moreover, SAT1 is markedly upregulated in allergic tissues, particularly in mast cells, where it promotes degranulation by suppressing autophagy. Mechanistically, SAT1 physically interacts with the long noncoding RNA Cdk19os to inhibit autophagy‐related genes, including *Atg5* and *Atg7*. Genetic or pharmacological targeting of the SAT1‐Cdk19os‐autophagy axis through SAT1/Cdk19os knockdown, spermidine supplementation, or autophagy induction effectively ameliorates allergic inflammation and reduces mast cell activation. These findings reveal that metabolic reprogramming actively drives food allergy development and identify tractable therapeutic targets and accessible dietary interventions in murine models. These results establish a conceptual framework for understanding the metabolic‐immunological basis of food allergy and open new avenues for translational investigation into this increasingly prevalent condition.

## Graphical abstract

Spermidine depletion, driven by upregulated spermidine/spermine N1‐acetyltransferase (SAT1), potentiates food allergy by promoting mast cell degranulation through autophagy suppression. SAT1 physically interacts with the long noncoding RNA Cdk19os to inhibit *Atg5* and *Atg7*, disrupting autophagic flux. Targeting the SAT1–Cdk19os–autophagy axis effectively attenuates allergic inflammation, identifying metabolic reprogramming as a tractable therapeutic strategy for food allergy.

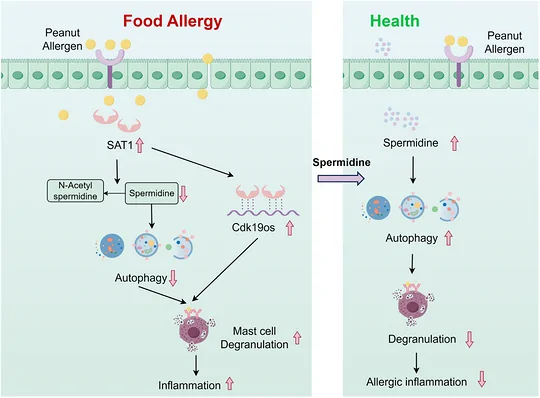

## 1 Introduction

Food allergies represent a growing global health crisis, affecting approximately 8% of children and 5% of adults worldwide, with the prevalence increasing 3.5‐fold over the past two decades [1, 2]. Among them, peanut allergy is particularly concerning due to its severe clinical manifestation, persistence into adulthood in approximately 75%–80% of affected children, and low threshold doses for triggering potentially fatal anaphylactic reactions [2, 3]. Despite this rising burden, disease‐modifying therapies remain critically limited, and current management strategies cannot reverse established allergic sensitization or tolerated for long [4, 5]. This gap highlights the urgent need to elucidate the molecular mechanisms underlying food allergy pathogenesis to enable the development of targeted interventions.

Food allergy responses are characterized by inappropriate Th2‐biased immune activation, leading to allergen‐specific immunoglobulin E (IgE) production and mast cell sensitization [6]. Upon allergen re‐exposure, IgE‐mediated mast cell degranulation leads to the release of inflammatory mediators, including histamines, proteases, and cytokines, precipitating immediate hypersensitivity reactions [6, 7]. However, the metabolic factors that initiate and sustain this immunological cascade remain incompletely understood, representing a critical knowledge gap in allergy research.

Emerging evidence suggests that cellular metabolism plays a pivotal role in shaping immune cell function and inflammatory responses, with metabolic reprogramming occurring in immune cells across various disease contexts [8, 9]. Polyamines, including putrescine, spermidine, and spermine, are small aliphatic polycations essential for cellular proliferation, differentiation, and survival [10]. Beyond their classical roles in cell growth, polyamines have recently emerged as immunomodulatory metabolites, with spermidine demonstrating anti‐inflammatory properties in various diseases [11, 12, 13, 14, 15]. Given the central role of dysregulated inflammation in allergic pathogenesis and the established anti‐inflammatory activities of polyamines, we hypothesized that polyamine metabolism might represent a critical metabolic checkpoint governing allergic responses.

Polyamine homeostasis is tightly controlled by biosynthetic and catabolic enzymes, including spermidine/spermine N^1^‐acetyltransferase (SAT1), the rate‐limiting enzyme in polyamine catabolism that converts spermidine and spermine into acetylated derivatives that are subsequently exported or further degraded [16, 17]. SAT1 expression is highly induced by various stimuli, including toxins, hormones, cytokines, and stress pathways, suggesting its potential involvement in various pathological processes [17]. However, the role of SAT1‐mediated polyamine catabolism in food allergy pathogenesis remains unclear.

Here, we report that spermidine depletion, driven by SAT1 upregulation, potentiates food allergies via a previously unrecognized mechanism. Using a peanut allergy model complemented by additional food allergen systems, we demonstrated that SAT1‐mediated spermidine catabolism promotes mast cell degranulation by suppressing autophagy. Mechanistically, we identified the long noncoding RNA (lncRNA) Cdk19os as a direct SAT1‐interacting partner that mediates autophagy inhibition and allergic effector functions. Targeting the SAT1‐Cdk19os‐autophagy axis through genetic or pharmacological approaches effectively ameliorated allergic responses, revealing the involvement of metabolic vulnerability in food allergy pathogenesis, with therapeutic implications.

## 2 Results

### 2.1 Spermidine Depletion Characterizes Food Allergy Pathogenesis

**FIGURE 1:**
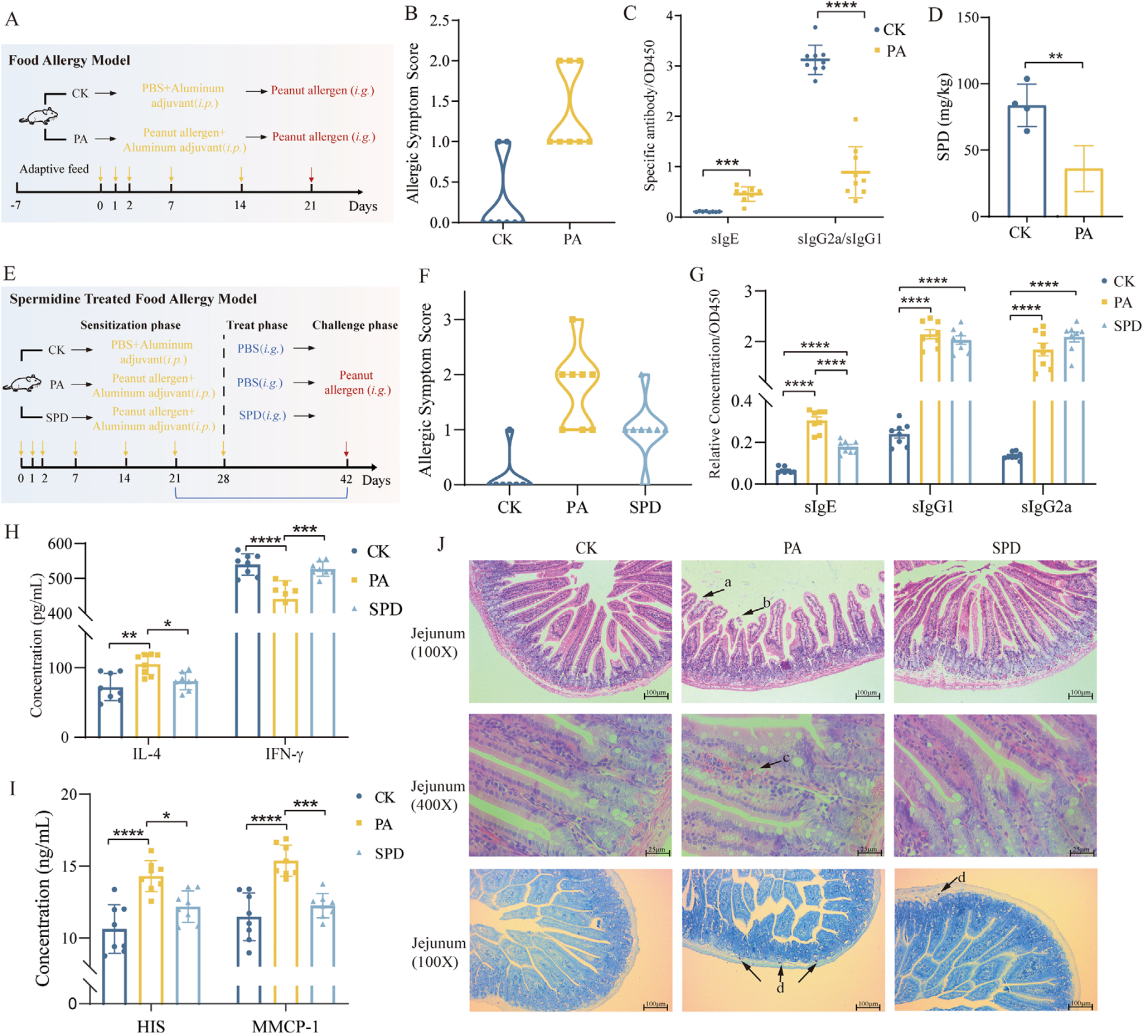
Spermidine depletion exacerbates peanut allergy susceptibility. (A) Schematic representation of the peanut allergy mouse model. BALB/c mice (*n* = 8) underwent five sensitizations with peanut protein combined with aluminum hydroxide adjuvant, followed by oral peanut protein challenge. (B) Clinical allergy scores assessed upon final challenge (*n* = 8). (C) Serum levels of peanut‐specific IgE (sIgE) and the sIgG2a/sIgG1 ratio (*n* = 8). (D) Jejunal spermidine (SPD) content quantified by high‐performance liquid chromatography‐mass spectrometry (HPLC‐MS) (*n* = 4). (E) Schematic representation of SPD supplementation in the peanut allergy model. BALB/c mice were sensitized with peanut protein, followed by daily oral gavage of SPD or vehicle control for 21 days before challenge (*n* = 8). (F) Clinical allergy scores upon final challenge (*n* = 8). (G) Serum levels of peanut‐specific IgE, IgG1, and IgG2a (*n* = 8). (H) Serum concentrations of IL‐4 and IFN‐γ (*n* = 8). (I) Serum concentrations of histamine and mouse mast cell protease‐1 (MMCP‐1) (*n* = 8). (J) Representative photomicrographs of H&E‐ and toluidine blue‐stained jejunal sections. Scale bar, 100 µm. Arrows indicate: (a) intestinal epithelial cell shedding; (b) villus shortening; (c) neutrophil infiltration; (d) mast cells. Data are presented as mean ± s.e.m. *p < 0.05, ** *p* < 0.01, *** *p* < 0.001, **** *p* < 0.0001.

To investigate the role of polyamine metabolism in food allergies, we established a peanut allergy model using BALB/c mice (Figure 1A). Following peanut allergen challenge, sensitized mice exhibited pronounced allergic responses, as evidenced by their elevated clinical allergy scores (Figure 1B); reduced body temperature, indicative of anaphylaxis (Figure S1A); and markedly increased serum allergen‐specific IgE levels (Figure 1C). These mice also showed increased sIgG1/sIgG2a ratios (Figure 1C) and a Th2‐biased cytokine profile characterized by elevated IL‐33 and IL‐4 alongside decreased IFN‐γ secretion (Figure S1B1), collectively confirming the establishment of Th2‐dominant immune responses characteristic of peanut allergy.

To assess polyamine status during allergic responses, we quantified putrescine, spermidine, and spermine in jejunal tissues using high‐performance liquid chromatography‐mass spectrometry (HPLC‐MS). Notably, spermidine levels were significantly reduced in allergic mice compared to those in control mice (Figure 1D, Figure S1C), while putrescine and spermine levels remained relatively stable, suggesting a spermidine‐specific association between polyamine metabolism and food allergy pathogenesis.

To establish whether spermidine depletion contributes to allergic pathology or merely represents an epiphenomenon, we supplemented mice with exogenous spermidine throughout the sensitization protocol. Prior to the main experiment, a dose‐finding study was conducted in which sensitized mice received spermidine at 5, 10, or 20 mg/kg; assessment of serum specific IgE and cytokine levels demonstrated that 10 mg/kg yielded the most pronounced reductions (Figure S2A, B). Accordingly, 10 mg/kg was selected for all subsequent in vivo experiments (Figure 1E). While body weight remained comparable across groups (Figure S2C), spermidine‐treated mice exhibited markedly attenuated allergic responses following the peanut challenge. Specifically, these mice displayed significantly reduced allergy symptom scores (Figure 1F), maintained their body temperatures (Figure S2D), and exhibited decreased allergen‐specific IgE levels (Figure 1G). Spermidine supplementation also reduced serum IgG1/IgG2a ratios (Figure 1G, Figure S2E) and shifted the cytokine profile away from Th2 polarization, as evidenced by decreased IL‐4 and IL‐33 levels alongside increased IFN‐γ and IL‐2 secretion (Figure 1H, Figure S2F). Moreover, spermidine treatment reduced intestinal inflammation, as evidenced by lower levels of histamine and mast cell protease‐1 (Figure 1I), and preserved jejunal villus architecture with reduced epithelial cell shedding (Figure 1J). These findings establish spermidine depletion as a pathogenic feature of peanut allergy and demonstrate that exogenous spermidine supplementation confers protection against both systemic and local allergic responses.

### 2.2 SAT1 Upregulation Drives Spermidine Depletion in Food Allergy

**FIGURE 2:**
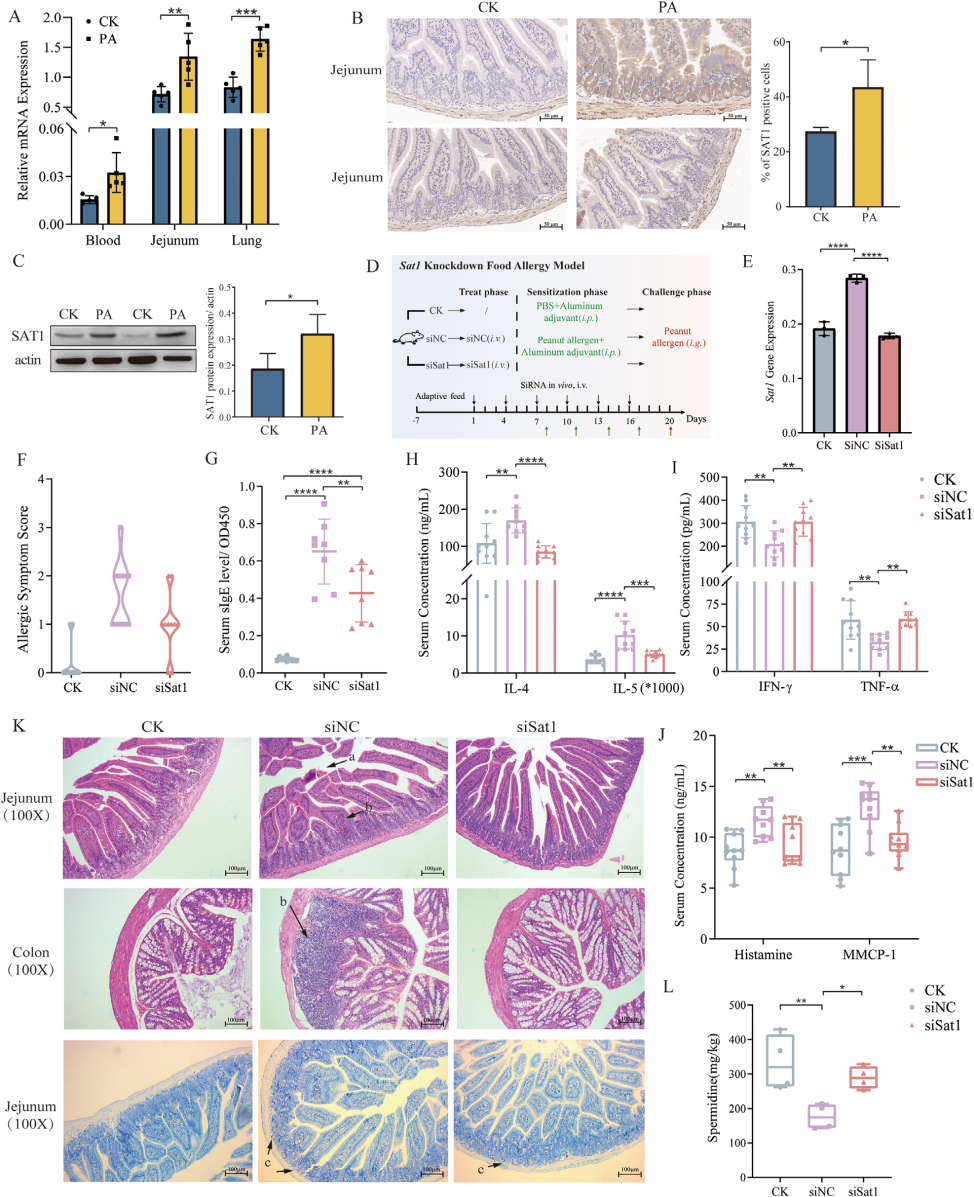
SAT1 upregulation mediates spermidine depletion and enhances peanut allergy susceptibility. (A) *Sat1* mRNA expression levels across multiple tissues (*n* = 5). (B) Representative immunohistochemistry images showing SAT1 protein localization in jejunal tissue (*n* = 3). Scale bar, 50 µm. (C) Representative western blot images of SAT1 protein in jejunal tissue. (D) Schematic representation of the *Sat1* knockdown peanut allergy model. BALB/c mice received tail vein injections of control or *Sat1*‐targeting siRNA, followed by peanut protein sensitization and challenge (*n* = 10). (E) Jejunal *Sat1* mRNA expression levels (*n* = 3). (F) Clinical allergy scores upon final challenge (*n* = 10). (G) Serum peanut‐specific IgE levels (*n* = 8). (H) Serum concentrations of the Th2‐type cytokines IL‐4 and IL‐5 (*n* = 8). (I) Serum concentrations of the Th1‐type cytokines IFN‐γ and TNF‐α (*n* = 10). (J) Serum concentrations of histamine and MMCP‐1 (*n* = 10). (K) Representative photomicrographs of H&E‐ and toluidine blue‐stained jejunal sections. Scale bar, 100 µm. Arrows indicate: (a) intestinal epithelial cell shedding; (b) neutrophil infiltration; (c) mast cells. (L) Jejunal spermidine content measured by HPLC‐MS (*n* = 4). Data are presented as mean ± s.e.m. *p < 0.05, **p < 0.01, ***p < 0.001, ****p < 0.0001.

Having demonstrated that spermidine depletion correlates with food allergy development, we sought to identify the underlying mechanisms. Since polyamine levels are tightly controlled by biosynthetic and catabolic enzymes, we hypothesized that dysregulation of these metabolic enzymes might account for the observed spermidine depletion. To test this, we first examined peripheral blood as a readily accessible biofluid that reflects systemic metabolic alterations during allergic responses. Analysis of polyamine metabolic enzymes in peripheral blood revealed that the mRNA levels of SAT1, the rate‐limiting enzyme in polyamine catabolism (Figure S3), were significantly elevated in allergic mice (Figure S4A). This upregulation extended to both the circulatory system and allergic effector organs, including the jejunum and lungs (Figure 2A). To further validate these findings, we reanalyzed a publicly available peanut allergy transcriptomic dataset (GSE17595), which corroborated the upregulation of SAT1 in allergic conditions (Figure S4B). Immunohistochemistry and immunohistochemistry western blotting analysis confirmed substantially increased SAT1 protein expression in allergic mice compared to that in control mice (Figure 2B,C), implicating SAT1 in peanut allergy pathogenesis. We further examined SAT1 expression in two additional food allergy models. Notably, *Sat1* levels were similarly elevated in the jejunal tissues of both whey protein‐allergic and shrimp‐allergic mice (Figure S4C), suggesting that SAT1 upregulation is a conserved feature of food allergy pathogenesis.

To establish causality, we generated a systemic *Sat1* knockdown model using in vivo siRNA delivery (Figure 2D). Following quantitative PCR (qPCR) verification of knockdown efficiency (Figure 2E) and confirmation of no systemic toxicity (Figure S4D), *Sat1*‐depleted mice exhibited markedly attenuated responses to the peanut challenge. These mice displayed reduced allergy symptom scores (Figure 2F), maintained their body temperatures (Figure S4E), and showed decreased allergen‐specific antibody levels (Figure 2G, Figure S4F,G). Immunologically, *Sat1* knockdown reversed allergen‐induced Th2 polarization, as demonstrated by reduced serum IL‐4 and IL‐5 (Figure 2H) and elevated IFN‐γ and TNF‐α secretion (Figure 2I).

The protective effects of *Sat1* knockdown extended to mast cell activation and tissue integrity. *Sat1*‐depleted mice showed reduced levels of histamine and mast cell protease‐1 (MMCP‐1) (Figure 2J), with decreased mast cell degranulation, as confirmed by toluidine blue staining of intestinal tissues (Figure 2K). Moreover, these mice exhibited preserved jejunal villus architecture and reduced inflammatory cell infiltration into colonic tissue (Figure 2K, Figure S4H). Critically, *Sat1* knockdown restored spermidine levels to those in control mice (Figure 2L), directly linking SAT1 activity to spermidine depletion. These findings establish that SAT1‐mediated spermidine catabolism is a critical mechanism underlying food allergy development.

### 2.3 SAT1 Promotes Allergic Responses through Mast Cell Degranulation

**FIGURE 3:**
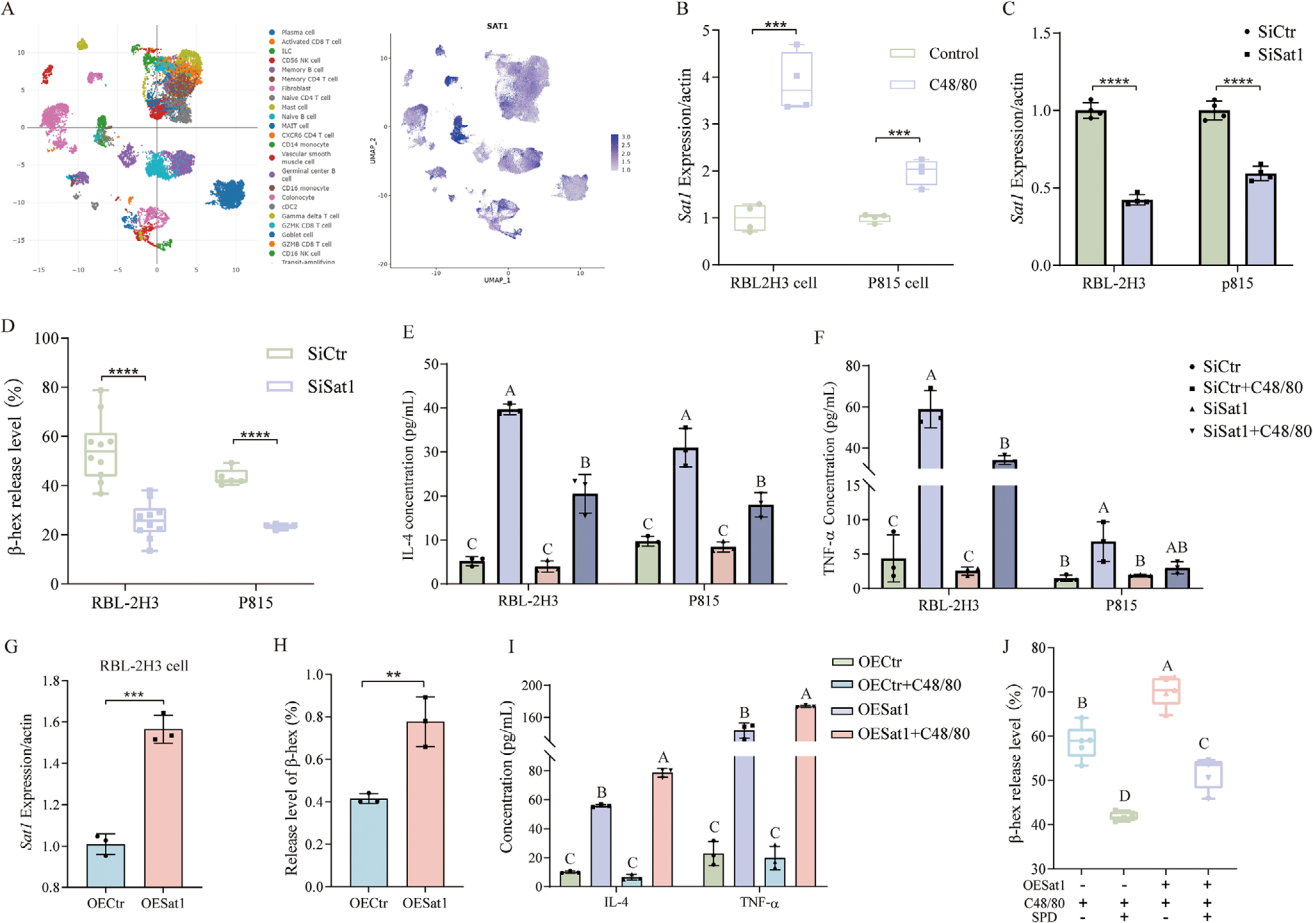
SAT1 enhances mast cell degranulation sensitivity. (A) SAT1 expression across distinct intestinal cell populations (data from DISCO database). (B) *Sat1* mRNA expression in RBL‐2H3 and P815 mast cells following compound 48/80 (C48/80) stimulation (*n* = 4). (C) *Sat1* mRNA expression in mast cells following siRNA‐mediated knockdown (*n* = 4). (D) β‐hexosaminidase release rate in mast cells following *Sat1* knockdown and C48/80 stimulation (*n* = 5/10). (E,F) Concentrations of IL‐4 and TNF‐α in cell culture supernatants after *Sat1* knockdown and C48/80 treatment (*n* = 3). (G) *Sat1* mRNA expression in RBL‐2H3 cells following lentiviral transduction (LV5 vector) (*n* = 3). (H) β‐hexosaminidase release rate in SAT1‐overexpressing cells following C48/80 stimulation (*n* = 3). (I) Concentrations of IL‐4 and TNF‐α in cell culture supernatants from SAT1‐overexpressing cells treated with C48/80 (*n* = 3). (J) β‐hexosaminidase release rate in SAT1‐overexpressing cells with or without spermidine supplementation (*n* = 5). Data are presented as mean ± s.e.m. *p < 0.05, ** *p* < 0.01, *** *p* < 0.001, **** *p* < 0.0001.

To identify the cellular targets of SAT1, we analyzed the cell type‐specific expression pattern of SAT1 in human intestinal single‐cell RNA sequencing databases. SAT1 expression was enriched in macrophages, neutrophils, and notably mast cells (Figure 3A), which are the key effector cells in food allergy that release inflammatory mediators, including histamines. This expression pattern was corroborated by single‐cell transcriptomic analysis of patients with allergic inflammation (Figure S5A), prompting an investigation into whether SAT1 regulates mast cell function.

Using compound 48/80 (C48/80)‐stimulated RBL‐2H3 (a rat‐derived basophilic leukemia cell line)and P815 (a murine mastocytoma cell line) as in vitro degranulation models (Figure S5B), we observed significantly elevated *Sat1* mRNA levels upon activation (Figure 3B). siRNA‐mediated *Sat1* knockdown reduced expression by 60% in RBL‐2H3 cells and 40% in P815 cells (Figure 3C), resulting in greater than 50% reduction in β‐hexosaminidase release (Figure 3D)—a canonical marker of mast cell degranulation. Moreover, *Sat1* knockdown markedly decreased secretion of the inflammatory cytokines IL‐4 and TNF‐α following C48/80 stimulation (Figure 3E,F). Conversely, lentiviral‐mediated *Sat1* overexpression in RBL‐2H3 cells (Figure 3G) significantly enhanced degranulation sensitivity, as evidenced by increased release of β‐hexosaminidase, IL‐4, and TNF‐α (Figure 3H,I). These complementary gain‐ and loss‐of‐function experiments established SAT1 as a positive regulator of mast cell degranulation.

Given that *Sat1* overexpression depletes spermidine, we investigated whether exogenous spermidine supplementation could counteract the enhanced degranulation. Spermidine exhibited concentration‐dependent effects on RBL‐2H3 cells: physiological concentrations (≤100 µM) inhibited degranulation, whereas supraphysiological concentrations paradoxically enhanced it, potentially through activation of compensatory metabolic feedback mechanisms (Figure S5C–F). Importantly, optimal spermidine supplementation effectively reversed the enhanced degranulation phenotype induced by *Sat1* overexpression (Figure 3J), demonstrating that SAT1 promotes allergic responses via spermidine‐dependent regulation of mast cell degranulation. To further assess whether spermidine modulates degranulation broadly, we examined the canonical IgE/FcεRI pathway. RBL‐2H3 cells sensitized with anti‐DNP IgE and challenged with DNP‐HSA exhibited FcεRI‐dependent degranulation. However, exogenous spermidine attenuated this response in a concentration‐dependent manner, an effect similarly observed in primary bone marrow‐derived mast cells (BMMCs)(Figure S5G,H). Together, these findings demonstrate that SAT1 promotes mast cell degranulation and allergic responses through spermidine depletion, with suppressive effects extending across both the MrgX2/C48/80 and IgE/FcεRI pathways.

### 2.4 SAT1 Inhibits Mast Cell Autophagy to Promote Degranulation

**FIGURE 4:**
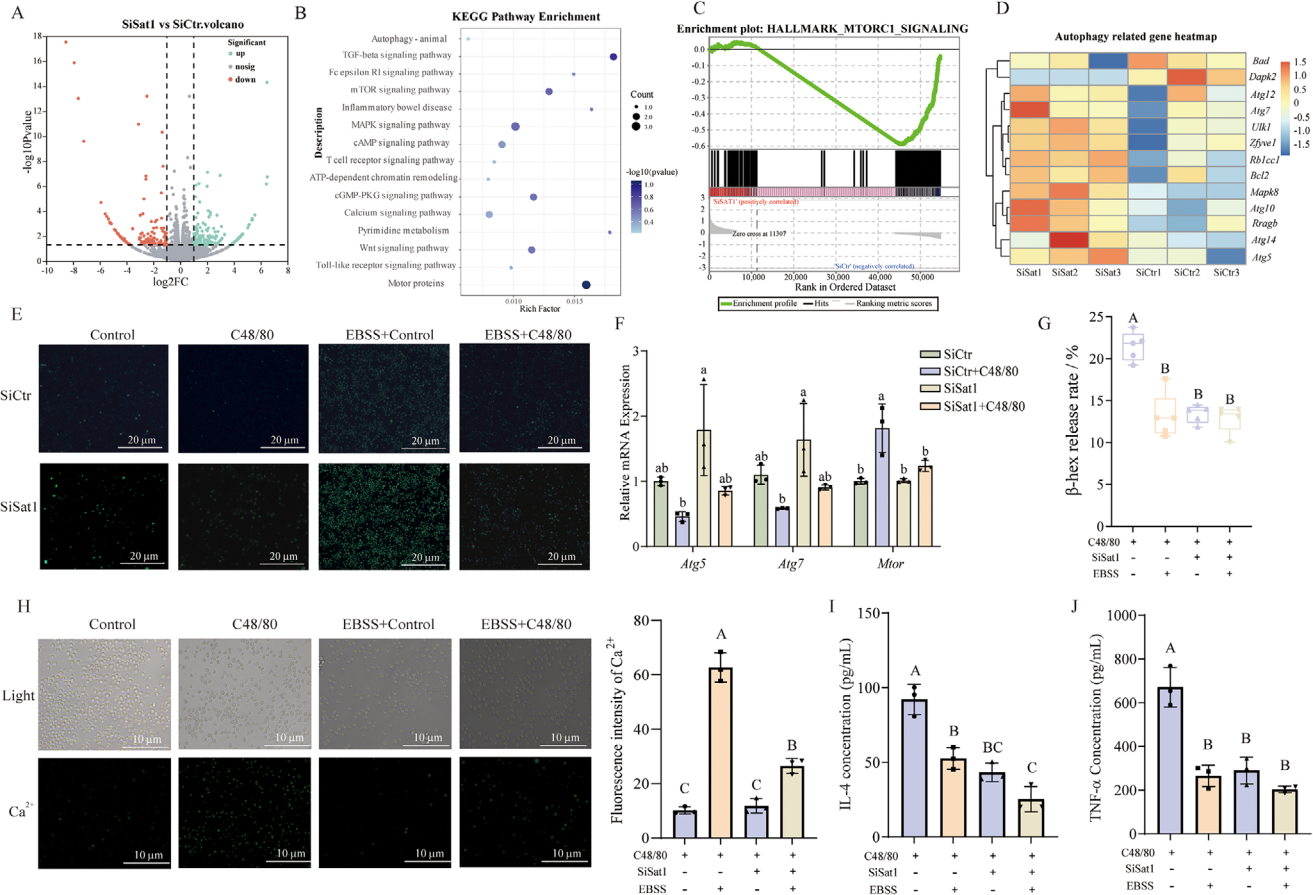
SAT1 suppresses mast cell autophagy. (A) Volcano plot of differentially expressed genes (DEGs) in C48/80‐treated P815 cells with or without *Sat1* knockdown. (B) Kyoto Encyclopedia of Genes and Genomes pathway enrichment analysis of DEGs. (C) Gene‐set enrichment analysis (GSEA) showing autophagy pathway enrichment. (D) Heatmap of autophagy‐related gene expression patterns. (E) Autophagic flux visualization by monodansylcadaverine (MDC) staining. (F) mRNA expression of autophagy‐related genes (*n* = 3). (G) β‐hexosaminidase release in P815 cells following autophagy activation by Earle's balanced salt solution (EBSS) (*n* = 3). Different letters denote statistical groupings based on compact letter display, where groups sharing at least one common letter are not significantly different (*P* ≥ 0.01), whereas groups without shared letters are significantly different (*p* < 0.01). (H) Intracellular Ca^2^^+^ levels in P815 cells following EBSS treatment (*n* = 3). (I,J) Secretion of IL‐4 and TNF‐α following autophagy activation (*n* = 3). Data are presented as mean ± s.e.m. *p < 0.05, ** *p* < 0.01, *** *p* < 0.001, **** *p* < 0.0001.

To elucidate the molecular mechanism by which SAT1 regulates mast cell degranulation, we performed transcriptomic profiling of P815 cells transfected with control or *Sat1* siRNA. *Sat1* knockdown resulted in 391 differentially expressed genes (235 upregulated and 156 downregulated; Figure 4A). These genes were significantly enriched in the autophagy and mTOR signaling pathways (Figure 4B, Figure S6A), which was corroborated by Gene Ontology (GO) analysis and validated by gene‐set enrichment analysis (Figure 4C). Hierarchical clustering of autophagy‐related genes clearly discriminated between control and *Sat1*‐depleted groups (Figure 4D), suggesting that SAT1 regulates mast cell degranulation by modulating the mTOR‐autophagy axis.

As mTOR signaling negatively regulates autophagy, we examined autophagic flux in mast cells using monodansylcadaverine (MDC) staining. C48/80 stimulation significantly reduced autophagosome formation, indicating autophagy suppression (Figure 4E, Figure S7A). Conversely, Earle's balanced salt solution (EBSS), a canonical autophagy inducer, substantially enhanced autophagic flux, which was blunted by concurrent C48/80 treatment. Notably, *Sat1* knockdown restored autophagy and synergized with EBSS treatment to enhance autophagic activity. Mechanistically, C48/80 treatment decreased the expression of the autophagy effectors *Atg5* and *Atg7* by approximately 50% while increasing *Mtor* expression in both P815 and RBL‐2H3 cells. However, these changes were reversed by *Sat1* knockdown (Figure 4F, Figure S6B,C).

To establish whether autophagy suppression directly mediates SAT1‐promoted degranulation, we pretreated P815 cells with EBSS prior to C48/80 stimulation. EBSS pretreatment significantly reduced β‐hexosaminidase release (Figure 4G), suppressed calcium influx (Figure 4H), and diminished IL‐4 and TNF‐α secretion (Figure 4I,J, Figure S7B,C), with an efficacy comparable to that of *Sat1* knockdown. These findings demonstrate that SAT1 enhances mast cell degranulation by suppressing autophagy, establishing a mechanistic link between polyamine metabolism, autophagy, and allergic effector cell function.

### 2.5 Autophagy Restoration Attenuates Food Allergy in Vivo

**FIGURE 5:**
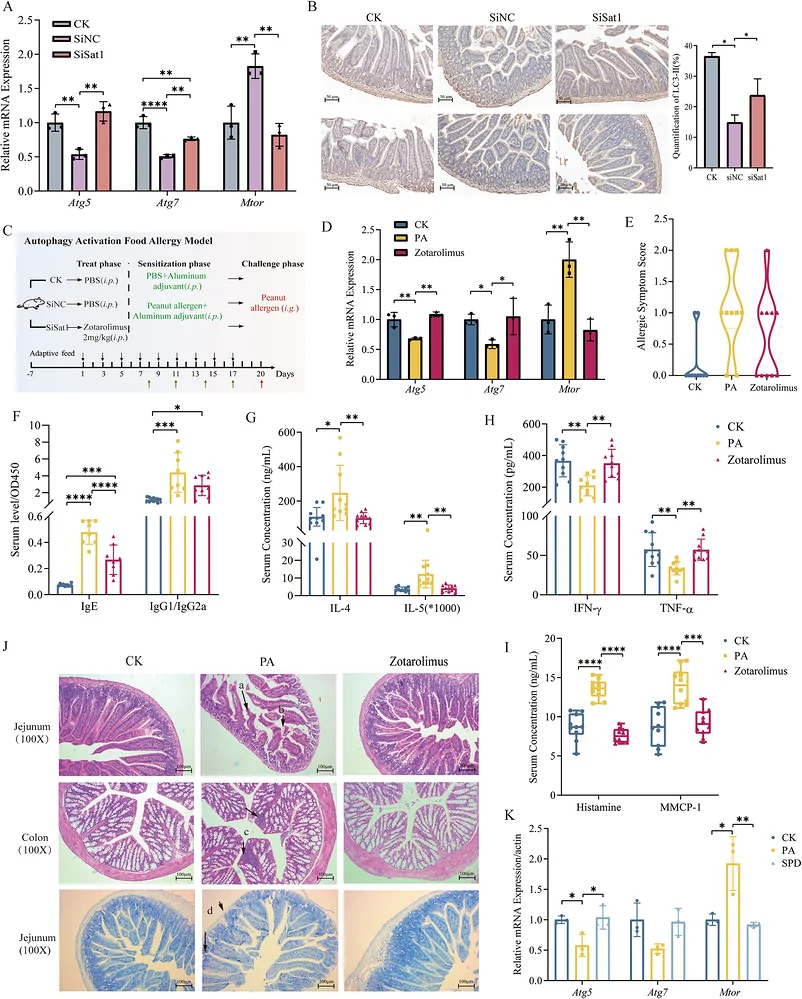
SAT1 promotes peanut allergy by suppressing autophagy. (A) mRNA expression of autophagy‐related genes in jejunal tissue from peanut‐allergic mice (*n* = 3). (B) Representative immunohistochemistry images and quantification of LC3‐II expression in jejunal tissue (*n* = 5). (C) Schematic representation of the autophagy activation model. CK, control mice; PA, peanut allergy model mice; Zotarolimus, PA mice treated with zotarolimus to activate autophagy (*n* = 10). (D) Jejunal mRNA expression of autophagy‐related genes (*n* = 3). (E) Clinical allergy scores upon final challenge (*n* = 10). (F) Serum peanut‐specific IgE levels and IgG1/IgG2a ratio (*n* = 10). (G) Serum concentrations of the Th2‐type cytokines IL‐4 and IL‐5 (*n* = 10). (H) Serum concentrations of the Th1‐type cytokines IFN‐γ and TNF‐α (*n* = 10). (I) Serum concentrations of histamine and MMCP‐1 (*n* = 10). (J) Representative photomicrographs of H&E‐ and toluidine blue‐stained jejunal sections. Scale bar, 100 µm. Arrows indicate: (a) villus shortening; (b) intestinal epithelial cell shedding; (c) neutrophil infiltration; (d) mast cells. (K) mRNA expression of autophagy‐related genes in jejunal tissue following SPD supplementation (*n* = 3). Data are presented as mean ± s.e.m. *p < 0.05, ** *p* < 0.01, *** *p* < 0.001, **** *p* < 0.0001.

To validate the pathogenic role of autophagy suppression in food allergies, we examined *Atg5* and *Atg7* expression in jejunal tissues of allergic mice. Both genes were significantly downregulated in sensitized animals, and this suppression was reversed by *Sat1* knockdown (Figure 5A, Figure S8A). Immunohistochemistry analysis of the autophagy marker LC3‐II corroborated these transcriptional changes (Figure 5B), confirming that SAT1 negatively regulates autophagy during allergic responses. To establish whether autophagy restoration was sufficient to ameliorate food allergy, we administered the autophagy inducer zotarolimus (Figure 5C). Treatment reversed allergen‐induced autophagy suppression, restoring *Atg5* and *Atg7* expression while reducing that of *Mtor* (Figure 5D, Figure S8B, C), without affecting body weight (Figure S8D). Autophagy activation attenuated allergic responses. Zotarolimus‐treated mice exhibited reduced symptom scores (Figure 5E), maintained their body temperatures (Figure S8E), and exhibited decreased allergen‐specific IgE and IgG1/IgG2a ratios (Figure 5F, Figure S8F). Moreover, the treatment suppressed Th2 cytokines (IL‐4 and IL‐5), while elevating Th1 cytokines (TNF‐α and IFN‐γ) (Figure 5G,H) and reduced mast cell activation markers (Figure 5I). Histologically, the mice displayed preserved jejunal architecture and reduced inflammation (Figure 5J, Figure S8G). Notably, spermidine supplementation upregulated autophagy genes (Figure 5K), suggesting that its protective effects were mediated through autophagy activation. These findings established autophagy as a critical node linking SAT1‐mediated spermidine depletion to allergic inflammation.

### 2.6 SAT1 Exerts Pro‐Allergic Effects through Cdk19os

To comprehensively elucidate the regulatory mechanism underlying SAT1‐mediated allergic pathogenesis, we investigated its molecular interactome. Emerging evidence indicates that metabolic enzymes can moonlight as RNA‐binding proteins to directly regulate gene expression [18], suggesting potential non‐catalytic functions beyond their established enzymatic activities. Computational analysis predicted a 65.2% probability of RNA‐binding activity for SAT1, raising the possibility that SAT1 might exert regulatory effects through direct RNA interactions in addition to its canonical role in polyamine catabolism.

**FIGURE 6:**
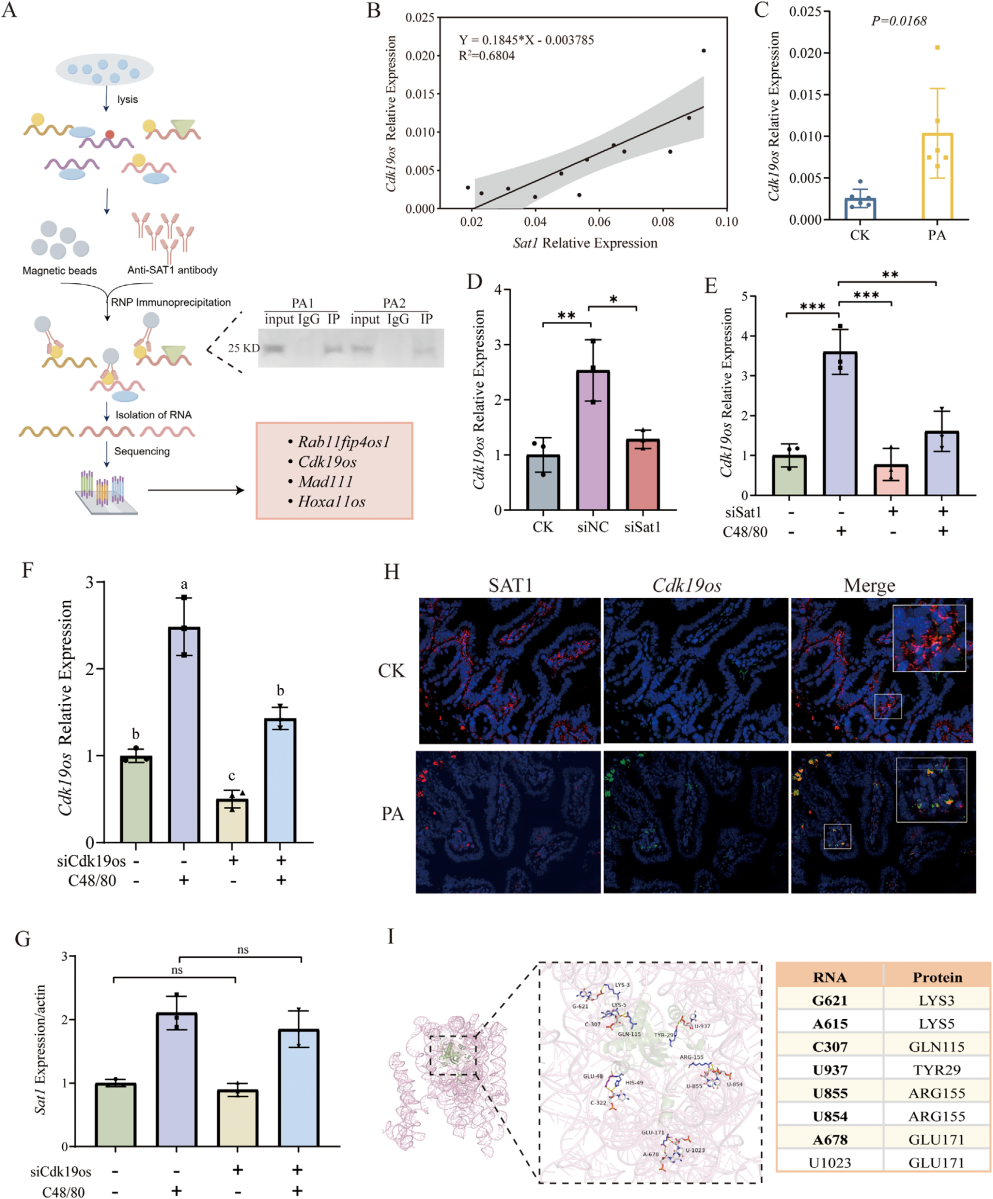
SAT1 interacts with the long noncoding RNA Cdk19os. (A) Schematic workflow of RNA immunoprecipitation sequencing (RIP‐Seq). (B) Correlation analysis between *Sat1* and *Cdk19os* expression. (C) Jejunal *Cdk19os* mRNA expression in peanut‐allergic mice (*n* = 5). (D) Jejunal *Cdk19os* expression in peanut‐allergic mice with or without *Sat1* knockdown (*n* = 3). (E) *Cdk19os* expression in P815 cells with or without *Sat1* knockdown (*n* = 3). (F) *Cdk19os* expression in P815 cells following siRNA‐mediated knockdown (*n* = 3). (G) *Sat1* mRNA expression in P815 cells following *Cdk19os* knockdown (*n* = 3). (H) Representative fluorescence in situ hybridization (FISH) images showing co‐localization of SAT1 (red) and Cdk19os (green); nuclei are stained with DAPI (blue). Scale bar, 50 µm. (I) Molecular docking simulation predicting SAT1‐Cdk19os interaction. Data are presented as mean ± s.e.m. *p < 0.05, ** *p* < 0.01, *** *p* < 0.001, **** *p* < 0.0001.

To identify SAT1‐interacting transcripts, we performed RNA immunoprecipitation sequencing (RIP‐Seq). SAT1 specifically bound to 841 lncRNAs with significant enrichment (fold‐enrichment >1, FDR <0.05), among which *Cdk19os* showed particularly strong interactions. In jejunal tissues, *Cdk19os* expression was strongly correlated with *Sat1* levels (R^2^ = 0.68, P = 0.001) and was upregulated 4.1‐fold in allergic mice (Figure 6A–C, Figure S9), suggesting that *Cdk19os* is a potential downstream effector of SAT1 in food allergies.

To establish the regulatory hierarchy between *Sat1* and Cdk19os, reciprocal knockdown experiments were performed. In Vivo, *Sat1* knockdown significantly suppressed *Cdk19os* expression in jejunal tissues (Figure 6D). Similarly, in C48/80‐stimulated P815 cells, *Sat1* knockdown reduced *Cdk19os* expression by 59% compared to that in controls (Figure 6E). Importantly, under basal conditions, *Cdk19os* levels were unaffected by *Sat1* depletion, indicating a stimulus‐dependent regulation. Conversely, *Cdk19os* knockdown did not alter Sat1 expression (Figure 6F,G), indicating that SAT1 is an upstream regulator. RNA fluorescence in situ hybridization (FISH) revealed extensive cytoplasmic co‐localization of Cdk19os and SAT1 (Figure 6H), suggesting direct physical interactions. Molecular docking simulations predicted a stable SAT1‐Cdk19os complex (docking score: −403.25) stabilized primarily by hydrogen bonding (Figure 6I). These data demonstrated that SAT1 regulates allergic inflammation by specifically binding to the lncRNA Cdk19os.

### 2.7 Cdk19os Inhibition Alleviates Food Allergy

**FIGURE 7:**
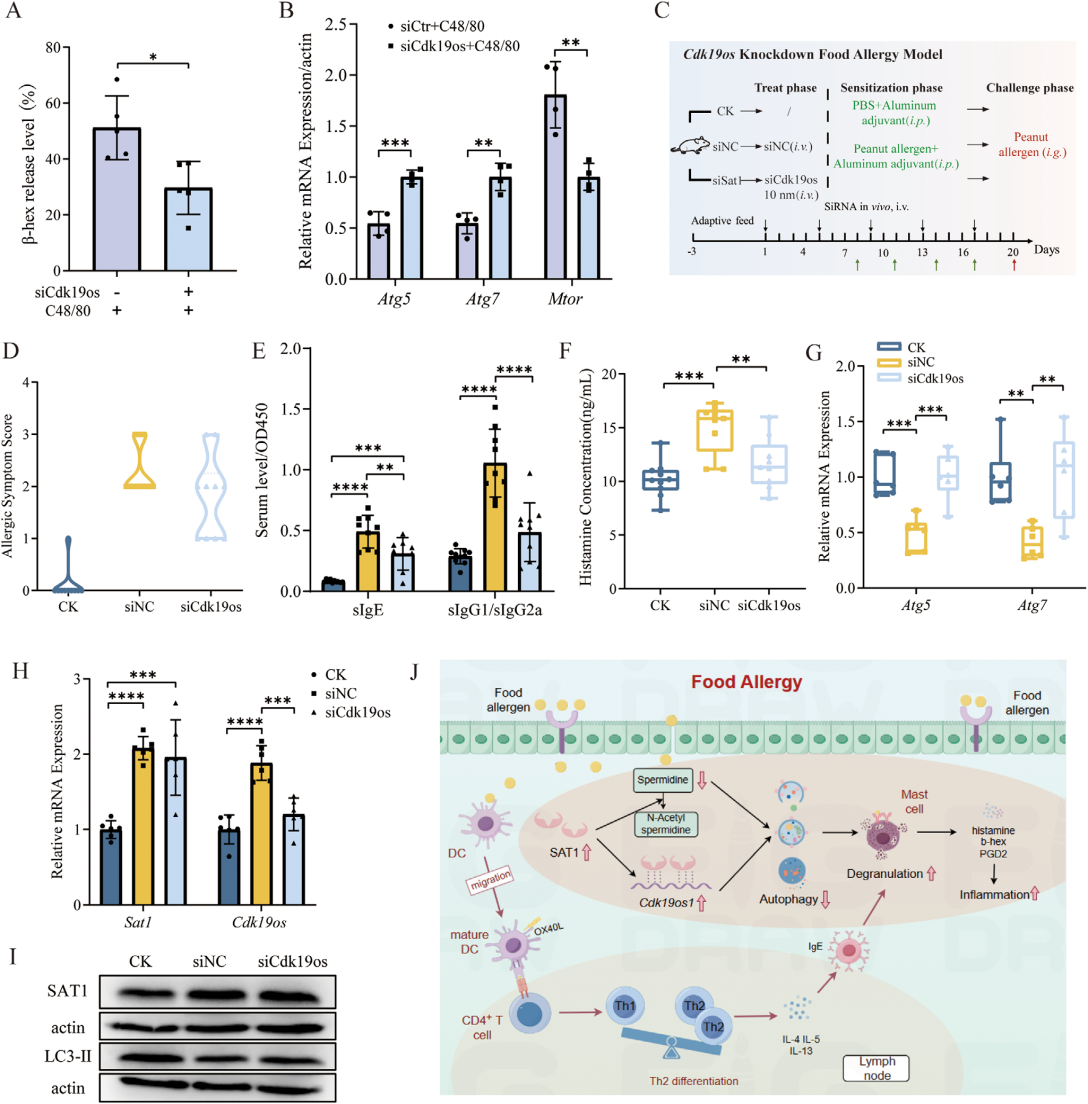
*Cdk19os* knockdown alleviates peanut allergy through autophagy activation. (A) β‐hexosaminidase release in P815 cells following siRNA‐mediated *Cdk19os* knockdown (*n* = 5). (B) mRNA expression of autophagy‐related genes following *Cdk19os* knockdown (*n* = 4). (C) Schematic representation of the *Cdk19os* knockdown peanut allergy model. BALB/c mice received tail vein injections of control or *Cdk19os*‐targeting siRNA, followed by peanut protein sensitization and challenge (*n* = 10). (D) Clinical allergy scores upon final challenge (*n* = 10). (E) Serum peanut‐specific IgE levels and IgG1/IgG2a ratio (*n* = 10). (F) Serum histamine concentration (*n* = 10). (G) Jejunal mRNA expression of autophagy‐related genes (*n* = 6). (H) Jejunal mRNA expression of *Sat1* and *Cdk19os* (*n* = 6). (I) Representative western blot images of SAT1 and LC3‐II protein in jejunal tissue. (J) Schematic diagram illustrating the SAT1–Cdk19os regulatory axis in peanut allergy pathogenesis. Data are presented as mean ± s.e.m. *p < 0.05, ** *p* < 0.01, *** *p* < 0.001, **** *p* < 0.0001.

To evaluate the therapeutic potential of targeting Cdk19os, we first assessed its functional role in mast cells using siRNA‐mediated knockdown. *Cdk19os* depletion significantly reduced β‐hexosaminidase release upon C48/80 stimulation (Figure 7A) and increased the expression of autophagy‐related genes (Figure 7B), indicating that Cdk19os promotes mast cell degranulation via autophagy suppression. We next established an *in vivo Cdk19os* knockdown model via tail vein siRNA delivery into allergic mice (Figure 7C). *In vivo Cdk19os* depletion (Figure 7C) attenuated allergic symptoms (Figure 7D, Figure S10A), decreased allergen‐specific IgE and IgG1/IgG2a ratios (Figure 7E), elevated IFN‐γ secretion (Figure S10B), and reduced serum histamine levels (Figure 7F). Mechanistically, *Cdk19os* knockdown restored autophagy in jejunal tissues, as evidenced by increased *Atg5* and *Atg7* expression, reduced *Mtor* levels (Figure 7G), and elevated LC3‐II protein levels (Figure 7H). Critically, *Sat1* expression remained unchanged following Cdk19os depletion at both mRNA and protein levels (Figure 7H,I), confirming the unidirectional SAT1‐Cdk19os regulatory axis. Collectively, these findings demonstrate that Cdk19os functions as a critical downstream effector of SAT1 in food allergy pathogenesis. Thus, the SAT1‐Cdk19os‐autophagy axis represents a novel regulatory pathway governing mast cell activation and allergic inflammation (Figure 7J).

## 3 Discussion

In this study, we identified a previously unrecognized metabolic‐immunological axis in food allergy pathogenesis that is centered on SAT1‐mediated spermidine depletion. Three novel and significant findings were obtained herein. First, spermidine depletion is not merely a bystander phenomenon but an active driver of allergic inflammation. Second, SAT1 suppresses mast cell autophagy, lowering the threshold for IgE‐mediated degranulation. And SAT1 operates as a moonlighting RNA‐binding protein that physically engages the lncRNA Cdk19os to enforce this autophagic block. Together, these findings delineate a SAT1–Cdk19os–autophagy regulatory axis with potential therapeutic relevance.

The finding that spermidine levels are selectively and significantly reduced in allergic tissues, while putrescine and spermine levels remain relatively stable, establishes a spermidine‐specific immunometabolic signature of food allergy. This finding substantially extends the emerging concept of immunometabolism into the realm of type 2 immune diseases, a context in which polyamine biology has not been previously investigated [6, 19, 20]. Prior work on spermidine has largely focused on its cardioprotective, neuroprotective, and longevity‐promoting properties, primarily attributed to its capacity to induce autophagy, effects of which have been documented in aging and chronic degenerative disease models; however, its relevance to IgE‐mediated allergic inflammation had not yet been explored [11, 15, 21]. Our finding that exogenous spermidine supplementation attenuates multiple hallmarks of peanut allergy (including systemic anaphylaxis, allergen‐specific IgE levels, Th2 cytokine profiles, and mast cell mediator release) while shifting the immune response toward Th1 dominance, is consistent with the established role of polyamine biosynthesis in supporting Th1 lineage fidelity [22]. These observations suggest that spermidine depletion creates a permissive metabolic environment for Th2‐biased allergic inflammation and that restoring polyamine homeostasis may represent an accessible dietary or pharmacological intervention strategy.

The preferential enrichment of SAT1 in intestinal mast cells of allergic animals offered a direct mechanistic link between polyamine catabolism and mast cell reactivity. SAT1 has previously been implicated in cancer progression and in sustained inflammatory states [23, 24], and its induction by TNF‐α, IL‐1β, and oxidative stress is consistent with a feed‐forward loop in which early allergic sensitization amplifies spermidine catabolism to further sustain the inflammatory milieu [25, 26]. Transcriptomic analysis revealed that SAT1 suppresses mTOR–autophagy signaling through downregulation of the core autophagy effectors *Atg5* and *Atg7*, positioning it upstream of an autophagic checkpoint that governs mast cell responsiveness. The effect of autophagy on mast cell activation is closely tied to its activity level, as basal autophagic flux is necessary for cellular homeostasis, yet its augmentation dampens degranulation by promoting granule content turnover and attenuating calcium mobilization [27, 28, 29]. The present findings extend this conceptual framework by showing that SAT1‐driven spermidine depletion erodes this autophagic brake, rendering mast cells hyperresponsive to allergen challenge. Notably, restoring autophagic flux, achieved either through Sat1 knockdown or pharmacological induction with zotarolimus, effectively suppressed degranulation and downstream allergic outcomes in vivo. Exogenous spermidine supplementation restored Atg5 and Atg7 expression in vivo, likely by replenishing the depleted polyamine pool and promoting autophagy through EP300 inhibition and calcineurin‐dependent TFEB nuclear translocation [30, 31, 32], thereby interrupting the vicious cycle between sustained SAT1 induction and impaired autophagic flux during allergic inflammation. Perhaps, the most mechanistically surprising finding of this study is that SAT1 exerts its regulatory effects not solely through its canonical enzymatic activity but also through direct physical interaction with the lncRNA Cdk19os. Computational prediction of RNA‐binding activity for SAT1, confirmed experimentally using RIP‐Seq, RNA FISH co‐localization, and molecular docking, revealed a non‐catalytic, moonlighting function for this metabolic enzyme [18, 33].

Metabolic enzymes serving as regulatory RNA‐binding proteins have attracted growing experimental attention in recent years [34, 35, 36], yet the demonstration that a polyamine catabolic enzyme interfaces with the noncoding transcriptome to modulate immune effector cell function constitutes a previously undescribed regulatory layer. Among the expanding catalog of lncRNAs implicated in immune cell biology [37, 38], Cdk19os had remained functionally uncharacterized prior to this work. The current data establish it as a critical effector downstream of SAT1, mediating autophagy suppression in mast cells. The functional necessity of Cdk19os in this pathway was confirmed by the concordant effects of its knockdown on *Atg5*/*Atg7* expression, LC3‐II flux, and in vivo allergic outcomes. SAT1 controls Cdk19os without detectable reciprocal feedback, which defines a coherent unidirectional signaling cascade linking a metabolic enzyme to a noncoding RNA intermediary and ultimately to the autophagy machinery. The precise molecular mechanism by which Cdk19os represses autophagy gene expression, be it through chromatin‐level scaffolding, competitive displacement of transcriptional activators, or post‐transcriptional regulation, remains an important open question for future work. We emphasize that the regulatory pathway mediated by Cdk19os has thus far been characterized in murine systems, and the existence of a human counterpart warrants further investigation.

Several limitations of the present study merit acknowledgment. At the mechanistic level, while our current data establish SAT1 as an upstream regulator of Cdk19os through convergent genetic, spatial, and computational evidence, the molecular basis by which Cdk19os suppresses Atg5/Atg7 expression has not been fully resolved in the present work. Formal epistatic validation will require future studies employing sequential or inducible genetic manipulation systems, such as Cdk19os overexpression in a stable SAT1‐knockdown background, to directly demonstrate rescue of mast cell degranulation. At the cellular level, because systemic siRNA delivery was used, definitive cell‐type‐specific attribution cannot be established; conditional deletion of Sat1 in mast cells will be essential to isolate their contribution from that of other stromal and hematopoietic populations. Although reanalysis of existing human transcriptomic datasets corroborates SAT1 upregulation in allergic patients, prospective studies are needed before clinical translation can be seriously pursued. Such studies should integrate SAT1 expression profiling, polyamine quantification, and autophagy marker assessment in well‐characterized patient cohorts that span diverse allergens and clinical phenotypes. Given the heterogeneity of human food allergies [39, 40], determining whether the SAT1‐Cdk19os axis varies across disease endotypes is important for personalized approaches.

Notwithstanding these limitations, the therapeutic opportunities suggested by this work are notable. Although oral immunotherapy is promising, its incomplete efficacy, long treatment duration, and safety concerns limit widespread application [41, 42]. Our findings that targeting the SAT1‐Cdk19os‐autophagy axis effectively ameliorates established allergic responses suggest an alternative therapeutic strategy. Multiple intervention nodes in the SAT1‐Cdk19os‐autophagy axis are pharmacologically accessible. First, autophagy inducers, such as rapamycin and zotarolimus, and their analogs, are FDA‐approved for immunosuppression and cancer treatment, [43] potentially facilitating clinical translation. Moreover, the recent development of SAT1 inhibitors for cancer therapy raises the possibility of repurposing these agents for allergic diseases [11, 15, 44]. Spermidine supplementation may be the most immediately translatable option, owing to its favorable safety profile, commercial availability, and established benefits in cardiovascular and aging‐related conditions [20]. Based on our preclinical findings, clinical trials testing spermidine supplementation in patients with food allergies are warranted after dose‐escalation studies in large animal models, together with systematic assessment of circulating spermidine levels in individuals.

In summary, this study establishes that SAT1‐mediated spermidine depletion is not a metabolic epiphenomenon of food allergy but an active driver of mast cell hyperresponsiveness. By connecting a classical metabolic enzyme to noncoding RNA biology and autophagic regulation, the findings reveal that SAT1–Cdk19os–autophagy pathway constitutes a mechanistically grounded therapeutic target. Spermidine depletion emerges as an immunometabolic hallmark of food allergy, lending support to the prospective evaluation of polyamine repletion through dietary supplementation or targeted pharmacotherapy as a viable preventive or adjunctive clinical strategy.

## 4 Methods

### 4.1 Mice and Ethics Statement

Female BALB/c mice (4–5 weeks old) were purchased from Beijing Vital River Laboratory Animal Technology Co., Ltd. (Beijing, China) and maintained under specific pathogen‐free conditions with a 12 h light/dark cycle at 22 ± 2°C and 50 ± 10% humidity. All animal procedures were approved by the Institutional Animal Care and Use Committee of China Agricultural University (Approval number: AW31214202‐5‐02) and performed in accordance with the Guide for the Care and Use of Laboratory Animals. Mice were monitored daily for signs of distress, including weight loss ≥20%, severe diarrhea, or irreversible morbidity, as predefined humane endpoints per institutional guidelines.

### 4.2 Animal Experimental Models

#### 4.2.1 Food Allergy Model

Mice were subjected to a peanut allergy induction protocol comprising sensitization and challenge phases. During sensitization (days 0, 1, 2, 7, and 14), mice in the peanut allergy group received intraperitoneal injections of 200 µL emulsion containing 2 mg/mL peanut protein complexed with aluminum hydroxide adjuvant (3:1, v/v), while controls received phosphate‐buffered saline (PBS). Following each injection, the abdominal area was gently massaged for 30 s to facilitate absorption. On day 21, sensitized mice were Orally challenged with 200 µL of 10‐fold concentrated peanut protein suspension.

#### 4.2.2 Spermidine Treatment Model

Mice were randomly assigned to control, peanut allergy, and spermidine groups (*n* = 10/group). During the sensitization phase (days 0, 1, 2, 7, 14, 21, and 28), the peanut allergy and spermidine groups received intraperitoneal injections of peanut protein extract, whereas the control group received only PBS. During days 21–41, the spermidine group received daily oral spermidine (10 mg/kg), while the control and peanut allergy groups received PBS. On day 42, all groups were orally challenged with a 10‐fold concentrated peanut protein suspension (200 µL).

#### 4.2.3 Knockdown Model

In Vivo knockdown of *Sat1* and *Cdk19os* was performed using chemically modified siRNAs (RiboBio Co., Guangzhou, China). The siRNA sequences (5'‐3') were as follows:

siSat1: sense strand, GGAAUGAACCAUCUAUCAA(dT)(dT); antisense strand, UUGAUAGAUGGUUCAUUCC(dT)(dT); siCdk19os: sense strand, GCUUUCUCGAAC UCAUUAA(dT)(dT); antisense strand, UUAAUGAGUUCGAGAAAGC(dT)(dT).

Mice received tail vein injections of siRNA (10 nmol) every three days. Sensitization was performed on days 8, 11, 14, and 17 using a peanut protein‐aluminum hydroxide mixture. On day 20, the mice were orally challenged with a 10‐fold concentrated peanut protein suspension to assess allergic response.

#### 4.2.4 Autophagy Activation Model

Mice were divided into control, peanut allergy, and autophagy‐activated (zotarolimus) groups (*n* = 10/group). The zotarolimus group received intraperitoneal injections of zotarolimus (2 mg/kg) every other day throughout the study. On days 8, 11, 14, and 17, the peanut allergy and zotarolimus groups were sensitized with peanut protein (1 mg/mL) in aluminum hydroxide (200 µL), while the control group received PBS. On day 20, all groups were orally challenged with concentrated peanut protein suspension (10 mg/mL, 200 µL).

The mice were monitored daily for survival, body weight changes, coat conditions, and spontaneous locomotor activity. At 30 min post‐challenge, allergic symptoms were scored as previously described, and blood samples were collected via retro‐orbital puncture under anesthesia. Mice were euthanized, and tissues (jejunum, colon, lung, and spleen) were either fixed in 4% paraformaldehyde for histology or snap‐frozen for molecular analysis.

### 4.3 Antibody and Cytokine Measurements

Blood samples were collected via retro‐orbital bleeding following challenge. Serum was separated by centrifugation at 3,000 × *g* for 15 min and stored at −80°C. Peanut‐specific IgE, IgG1, and IgG2a levels were measured using enzyme‐linked immunosorbent assay (ELISA) as previously described [45]. Serum concentrations of IL‐4, IL‐2, IL‐5, IL‐33, TNF‐α, and IFN‐γ were quantified using commercial ELISA kits according to the manufacturer's instructions (Multiscience, Hangzhou, China). Histamine and MMCP‐1 levels were determined using ELISA kits (Dogesce, Beijing, China). All samples were measured in duplicate, and absorbance was read at 450 nm using a microplate reader (Thermo Fisher Scientific, MA, USA).

### 4.4 Polyamine Quantification

Jejunal tissue samples (approximately 50 mg) were homogenized in ice‐cold ultrapure water and subjected to ultrasonic extraction for 30 min. Acetonitrile was then added, and the mixture was vortexed and sonicated for an additional 10 min. After centrifugation at 6,000 × *g* for 5 min at 4°C, the supernatant was filtered through a 0.22 µm membrane and analyzed by HPLC‐mass spectrometry using an Agilent C18 column (2.1 × 100 mm, 3 µm, Santa Clara, CA, USA) at 35°C with a flow rate of 0.3 mL/min and an injection volume of 5 µL. Mass spectrometry was performed in the negative electrospray ionization mode. Putrescine, spermidine, and spermine levels were quantified using external calibration curves prepared using authentic standards.

### 4.5 Histopathological Analysis

Jejunal and colonic tissues were fixed in 4% paraformaldehyde for 24 h at room temperature, dehydrated through a graded ethanol series, embedded in paraffin, and sectioned at 5 µm thickness using a microtome. Sections were deparaffinized and stained with hematoxylin and eosin for general morphological assessment or with toluidine blue O for mast cell identification. Histological parameters, including villus height, crypt depth, epithelial integrity, and inflammatory cell infiltration, were evaluated using light microscopy. Mast cells, identified by their characteristic metachromatic granules appearing as fuchsia‐stained cells, were quantified by counting fuchsia spots in each scanned field. Tissue sections were stained and analyzed in a blinded manner.

### 4.6 qPCR

Total RNA was extracted using the TRIzol reagent (Invitrogen, Carlsbad, CA, USA) according to the manufacturer's instructions and quantified using Nanodrop (Allsheng, Hangzhou, China). First‐strand cDNA was synthesized from RNA using the PrimeScript RT Reagent Kit with gDNA Eraser (Vazyme, Nanjing, China). qPCR was performed using ChamQ Universal SYBR qPCR Master Mix (Vazyme) on a CFX96 Real‐Time PCR Detection System (Bio‐Rad, Hercules, CA, USA). The thermal cycling conditions consisted of an initial denaturation at 95°C for 30 s, followed by 40 cycles at 95°C for 10 s, and 60°C for 30 s. Primer sequences were listed in Table S1. Relative mRNA expression levels were normalized to *Actin* expression levels and calculated using the 2^−ΔΔCt^ method. Each experiment was performed with at least three biological replicates.

### 4.7 Cell Experiments

#### 4.7.1 Cell Culture and Maintenance

RBL‐2H3 rat basophilic leukemia cells and P815 mouse mastocytoma cells were obtained from the National Biomedical Cell Resource Bank. RBL‐2H3 and P815 cells were cultured in minimum essential medium and Dulbecco's modified Eagle's medium, respectively, each supplemented with 10% fetal bovine serum and 1% penicillin‐streptomycin.

Generation of BMMCs was performed by isolating bone marrow cells from femurs/tibias of 6‐week‐old female BALB/c mice as previously described [46]. Briefly, bone marrow cells were flushed with complete RPMI‐1640 medium (supplemented with 10% fetal bovine serum, 1% penicillin‐streptomycin, 50 µM β‐mercaptoethanol, and 10 mM HEPES) and cultured at 37°C in a humidified 5% CO_2_ atmosphere in the presence of recombinant murine IL‐3 (10 ng/mL, PeproTech) and stem cell factor (10 ng/mL, PeproTech). Non‐adherent cells were collected and transferred to new flasks with fresh cytokine‐supplemented medium every 3–4 days. After 4–6 weeks of culture, mast cell purity was confirmed by flow cytometric analysis of c‐Kit (CD117) and FcεRI surface expression; only cultures consisting of >95% c‐Kit^+^ FcεRI^+^ cells were used for subsequent experiments.

#### 4.7.2 siRNA Transfection

For *Sat1* knockdown, cells were transfected with siRNA targeting mouse *Sat1* (sense: 5'‐GCAUCUACGAGAACUACAU‐3'; antisense: 5'‐AUGUAGUUCUCGUAGAUGC‐3') or scrambled control siRNA (GenePharma, Shanghai, China) according to the manufacturer's protocol. For *Cdk19os* knockdown, cells were transfected with siRNA targeting mouse *Cdk19os* (sense: 5'‐GCUUUCUCGAACUCAUUAA‐3'; antisense: 5'‐UUAAUGAGUUCGAGAAAGC‐3'). Knockdown efficiency was verified using qRT‐PCR 48 h post‐transfection.

#### 4.7.3 Lentiviral Overexpression

For *Sat1* overexpression experiments, lentiviral vectors (LV5‐OESat1 and empty vector control) were constructed by Suzhou Gemma Genes Co., Ltd. RBL‐2H3 cells (1 × 10^5^ cells/well in 12‐well plates) were infected with lentivirus (10^7^ transducing units/mL) in the presence of polybrene (5 µg/mL). After 24 h, the virus‐containing medium was replaced with a fresh medium. Overexpression efficiency was verified using qRT‐PCR after 48 h.

#### 4.7.4 Spermidine Treatment

Cells were seeded at 10^5^ cells/mL in 96‐well plates and cultured until 70% confluence. Cells were pretreated with various concentrations of spermidine diluted in minimum essential medium for 12, 24, 36, or 48 h, followed by stimulation with C48/80 or IgE. Degranulation was assessed by noting β‐hexosaminidase release.

#### 4.7.5 Autophagy Modulation

Autophagy was induced by replacing the complete culture medium with EBSS (Beyotime, Shanghai, China) for 4 h prior to subsequent experimental procedures.

#### 4.7.6 Cell Viability Assay

A total of 5 × 10^4^ cells were seeded onto a 96‐well plate and cultured overnight at 37°C. Cell viability was assessed using CCK‐8 reagent (Biorigin, Beijing, China) following different treatments according to the manufacturer's instructions.

#### 4.7.7 C48/80‐Mediated Mast Cell Activation and Degranulation Assay

RBL‐2H3 and P815 cells (5 × 10^6^ cells/mL, 200 µL/well) were seeded into 96‐well plates for 24 h. After washing with Tyrode's buffer, cells were stimulated with 100 µL C48/80 at varying concentrations for 1.5 h at 37°C. Tyrode's buffer and Triton X‐100 served as the negative and positive controls, respectively. The reaction was terminated by cooling on ice for 15 min. For β‐hexosaminidase quantification, 30 µL supernatant was incubated with 50 µL of 1.3 mg/mL p‐nitrophenyl‐N‐acetyl‐β‐D‐glucosaminide in citrate buffer. Reactions were stopped with 200 µL sodium carbonate buffer, and absorbance was measured at 405 nm. The supernatants were retained for cytokine analysis.

#### 4.7.8 IgE/FcεRI‐Mediated Mast Cell Degranulation Assay

After seeded, cells were sensitized by incubation with monoclonal anti‐dinitrophenyl (DNP) IgE antibody (1 µg/mL, Sigma‐Aldrich) in serum‐free DMEM for 12 h at 37°C. Following sensitization, cells were washed twice with Tyrode's buffer to remove unbound IgE, then challenged with DNP‐conjugated human serum albumin (DNP‐HSA; 100 ng/mL; Sigma‐Aldrich) in 100 µL Tyrode's buffer for 1 h at 37°C to trigger FcεRI‐dependent degranulation. The reaction was terminated by cooling on ice for 15 min. β‐hexosaminidase release was quantified as described in Section 4.7.7.

### 4.8 Immunohistochemical and Immunofluorescence Analyses

#### 4.8.1 Immunohistochemistry Staining

Paraffin‐embedded jejunal tissue sections (4 µm) were deparaffinized, rehydrated, and subjected to heat‐induced epitope retrieval using citrate buffer (pH 6.0). After blocking with 3% bovine serum albumin, sections were incubated overnight at 4°C with primary antibodies against SAT1 (1:500) or LC3II (1:500), followed by HRP‐conjugated secondary antibody (1:200). Immunoreactivity was visualized using DAB substrate with hematoxylin counterstaining. Images were captured using a light microscope (NIKON E100, Tokyo, Japan), and immunopositive areas were quantified as the average optical density using ImageJ (v1.53q, National Institutes of Health, Bethesda, MD, USA).

#### 4.8.2 Autophagy Flux Assessment

The autophagic activity of P815 cells was evaluated using MDC staining. Following experimental treatment, cells were incubated with 1 × MDC solution at 37°C for 30 min in darkness, washed three times, and immediately imaged by fluorescence microscopy (Olympus IX71, Tokyo, Japan) at 335/512 nm.

#### 4.8.3 Intracellular Calcium Measurement

Intracellular calcium concentrations were measured using the Fluo‐3 AM fluorescent probe (5 µM, Beyotime). Cells were loaded with the probe for 45 min at 37°C in the dark, washed to remove extracellular probe, and incubated for an additional 30 min to allow complete de‐esterification. Fluorescence images were captured using a fluorescence microscope (Olympus IX71; Tokyo, Japan).

#### 4.8.4 RNA FISH

Mouse jejunal tissues were fixed in FISH fixative at 4°C for 12–24 h, processed through graded ethanol/xylene, and paraffin‐embedded. Sections (4 µm) were deparaffinized and subjected to antigen retrieval in citrate buffer (10 mM, pH 6.0) at 95°C for 48 min. Following proteinase K digestion (40°C, 10 min) and pre‐hybridization (37°C, 1 h), the sections were hybridized overnight at 40°C with specific probes (Table S2). Post‐hybridization washes were performed with 2×, 1×, 0.5×, and 0.1× SSC at 40°C (5 min each). Signal amplification involved two rounds of amplification probe incubation (3 h each at 40°C) with intervening washes. Sections were counterstained with DAPI (1 µg/mL, 8 min), mounted with anti‐fade medium, and imaged using a fluorescence microscope (NIKON ECLIPSE CI).

### 4.9 Western Blotting

Jejunal samples were abraded and lysed in RIPA buffer supplemented with protease and phosphatase inhibitor cocktails (Beyotime, Shanghai, China). Protein concentrations were determined using the BCA. Equal amounts of protein lysates were resolved on SDS‐polyacrylamide gels and transferred to polyvinylidene difluoride membranes (Millipore, Bedford, MA, USA) using standard electrophoretic techniques. Membranes were blocked with 5% non‐fat milk and incubated overnight at 4°C with primary antibodies: anti‐SAT1 (1:1000, Invitrogen), anti‐LC3B (1:1000, Beyotime), and anti‐β‐actin (1:1000, Biorigin). After washing, the membranes were incubated with HRP‐conjugated secondary antibodies (1:10000; Beyotime) for 1 h at room temperature. Immunoreactive bands were visualized using enhanced chemiluminescence substrate (Millipore) and detected using a gel imaging system. Densitometric analysis was performed using ImageJ software, with β‐actin serving as the normalization control.

### 4.10 RNA Sequencing

Total RNA was extracted from P815 cells (*n* = 3 per group) using TRIzol reagent (Thermo Fisher Scientific), and RNA integrity was assessed using an Agilent 2100 Bioanalyzer. Samples with RNA integrity number ≥ 8 were used for library preparation. RNA sequencing libraries were constructed using the NEBNext Ultra RNA Library Prep Kit for Illumina (New England Biolabs, Ipswich, MA, USA), following the manufacturer's protocol, and sequenced on an Illumina NovaSeq 6000 platform (Illumina, San Diego, CA, USA), generating 150‐bp paired‐end reads. Library construction and sequencing were performed by Shanghai Meiji Biomedical Technology Co., Ltd.

Raw sequencing reads were quality‐trimmed using Trimmomatic v0.39 to remove adapter sequences and low‐quality bases. High‐quality reads were then aligned to the mouse reference genome (GRCm38/mm10) using HISAT2 v2.2.1, with default parameters. Read counts for each gene were quantified using featureCounts v2.0.1 from the Subread package. Differential gene expression analysis was performed using DESeq2 v1.30.0. Genes with |log_2_(fold change)| > 1 and adjusted P‐value < 0.05 (Benjamini–Hochberg correction) were considered differentially expressed. GO enrichment and Kyoto Encyclopedia of Genes and Genomes pathway analyses were conducted using clusterProfiler v3.18.0.

### 4.11 RIP‐Seq

To identify lncRNAs interacting with SAT1, mouse jejunal tissues were mechanically dissociated and lysed in RIP lysis buffer for 25 min on ice. Lysates (2 mg protein) were incubated with 5 µg SAT1 antibody or IgG control for 2 h at 4°C and then overnight with Protein A/G magnetic beads (Thermo Fisher Scientific). After washing with RIP wash buffers, complexes were eluted with 1% trifluoroacetic acid at 37°C and neutralized. RNA was extracted using TRIzol reagent and assessed using NanoDrop ND‐1000 and gel electrophoresis.

IP RNA (Cy5‐labeled) and input RNA (Cy3‐labeled) were prepared using an Arraystar RNA Labeling Kit and hybridized to Mouse RIP lncRNA/mRNA Expression Microarrays (8 × 60 K; Arraystar). The arrays were scanned using an Agilent G2505C scanner and analyzed using Feature Extraction v11.0.1. Data were normalized by log transformation with a median intensity correction. Microarray services were provided by Shanghai Genechem Biotechnology Co., Ltd.

### 4.12 Molecular Docking Analysis

The crystal structure of SAT1 (UniProt ID: P48026) was retrieved from UniProt and optimized using ChimeraX v1.7 to remove water molecules, heteroatoms, and non‐essential ligands. The target lncRNA sequence (GenBank: NR_190328.1) was obtained from NCBI, and its three‐dimensional structure was predicted using AlphaFold3, selecting the highest‐confidence model. Rigid‐flexible hybrid molecular docking was performed using the HDOCK server (http://hdock.phys.hust.edu.cn/), and the complex with the lowest docking score was selected as the optimal binding conformation. Molecular interactions, including hydrogen bonds, hydrophobic contacts, and electrostatic complementarity, were analyzed and visualized using PyMOL v3.0.3.

### 4.13 Statistical Analysis

Data were presented as mean ± standard error of the mean (s.e.m) from at least three independent biological replicates. Statistical analyses were performed using GraphPad Prism v9.0 (GraphPad Software, San Diego, CA, USA). For comparisons between two groups, a two‐tailed Student's *t*‐test was used. For multiple group comparisons, a one‐way analysis of variance (ANOVA) followed by Tukey's post‐hoc test was applied. Two‐way ANOVA was used for experiments with two independent variables. Pearson's correlation analysis was performed to assess the relationship between SAT1 and lncRNA expression. Protein‐RNA interaction predictions were conducted using catRAPID (http://service.tartaglialab.com/page/catrapid_group). Statistical significance was defined as *p* < 0.05, with *p < 0.05, **p < 0.01, and ***p < 0.001, as indicated in the figures. Sample sizes were determined based on preliminary experiments and prior publications to ensure adequate statistical power (≥0.80) for detecting biologically meaningful differences.

## Author Contributions

Conceptualization: H.L.C. and M.M.L.; Methodology: M.M.L., B.Y.Z., J.J.W., J.Y.; Investigation: M.M.L., S.W.H.; Validation: L.Y.Z.; Writing – original draft: M.M.L., J.F.W.; Writing – review & editing: M.M.L., J.F.W., H.L.C.; Resource: Q.W.W.; Project administration: J.F.W., H.L.C.; Funding acquisition: H.L.C.

## Conflicts of Interest

The authors declare no conflicts of interest.

## Supporting information




**Supporting File**: advs77798‐sup‐0001‐SuppMat.docx.

## Data Availability

The raw data of RNA sequencing in the current study were deposited at NCBI BioProject, and the accession number is PRJNA1440126.
