# Supplementary material for: SAT1‐Induced Spermidine Depletion Potentiates Food Allergy
Source: Adv Sci (Weinh). 2026 Sep 27:e77798. Online ahead of print. doi: 10.1002/advs.77798 (PMC13616371; doi:10.1002/advs.77798)
Supplement: Supplementary file 1 [file ADVS-9999-e77798-s001.docx]

Supporting Information

SAT1-induced Spermidine Depletion Potentiates Food Allergy

*Manman Liu, Beiyu Zhang, Junjuan Wang, Yue Jiang, Shiwen Han, Qianwei Wang, Lingyu Zeng, Jinfeng Wang***, Huilian Che**

**Figure S1. Spermidine levels in peanut-allergic mice.** **A** Rectal temperature changes following final challenge (n = 8). **B** Serum concentrations of IL-33, IL-4, and IFN-γ (n = 6). **C** Jejunal polyamine contents, including putrescine, spermine, and spermidine, measured by LC-MS (n = 4).

**Figure S2. Spermidine depletion enhances allergic responses in peanut-allergic mice. A** Effects of varying spermidine concentrations on serum peanut-specific antibody levels (n=5). **B** Effects of varying spermidine concentrations on serum IL-4 and IFN-γ levels (n = 5). Different letters denote statistical groupings based on compact letter display, where groups sharing at least one common letter are not significantly different (P ≥ 0.01), whereas groups without shared letters are significantly different (P < 0.01). **C** Body weight changes throughout the experimental period (n = 8). **D** Rectal temperature changes after final challenge in mice with or without SPD supplementation (n = 8). **E** Serum levels of sIgG2a/sIgG1 ratio (n = 8). **F** Serum concentrations of IL-33 and IL-2 (n = 6). Data are presented as mean ± s.e.m. *P < 0.05, ** P < 0.01, *** P < 0.001, **** P < 0.0001.

**Figure S3. Schematic representation of polyamine metabolic pathways.**

**Figure S4. Sat1 knockdown attenuates peanut allergy phenotypes.** **A** Gene expression levels of polyamine metabolic enzymes in peripheral blood (n = 3). **B** Temporal expression pattern of Sat1 from 0 to 120 min following peanut allergen challenge in CC027/GeniUnc mice (data from GSE179595 dataset). **C** Sat1 mRNA expression levels in allergic mice (n=5). **D** Body weight changes throughout the experimental period (n = 10). **E** Rectal temperature changes after final challenge in mice treated with control or Sat1-targeting siRNA (n = 10). **F**,**G** Serum peanut-specific IgG1 and IgG2a levels (n = 8). **H** Representative photomicrographs of H&E- and toluidine blue-stained colonic sections. Scale bar, 100 µm. Data are presented as mean ± s.e.m. *P < 0.05, ** P < 0.01, *** P < 0.001, **** P < 0.0001.

**Figure S5.** **Spermidine supplementation** **rescues SAT1-mediated mast cell hyperreactivity. A** SAT1 expression across different cell types (data from PMC6133715 dataset). **B** Dose-dependent β-hexosaminidase release induced by C48/80 in RBL-2H3 cells (n = 5). **C** Effect of varying spermidine concentrations on RBL-2H3 cell viability assessed by CCK-8 assay (n = 6). **D** Effect of varying spermidine concentrations on mast cell degranulation (n = 6). **E, F** Time-dependent effects of 10 mM spermidine on RBL-2H3 cell viability and degranulation (n = 6). **G, H** Effect of spermidine on IgE/FcεRI-mediated RBL-2H3 (G) and BMMC (H) cell degranulation (n = 6). Data are presented as mean ± s.e.m. *P < 0.05.

**Figure S6. *Sat1* knockdown promotes autophagy in mast cells. A** Gene Ontology (GO) enrichment analysis of DEGs. **B** Expression of autophagy-related genes in C48/80-treated P815 cells (n = 3). **C** Expression of autophagy-related genes following Sat1 knockdown (n = 3). Data are presented as mean ± s.e.m. *P < 0.05, ** P < 0.01, *** P < 0.001, **** P < 0.0001.

**Figure S7. Autophagy activation suppresses mast cell inflammatory responses**. **A** The quantification of mean fluorescence density of MDC (n = 3). **B,C** Secretion of IL-4 and TNF-α under different experimental conditions (n = 3). Data are presented as mean ± s.e.m. Different letters denote statistical groupings based on compact letter display, where groups sharing at least one common letter are not significantly different (P ≥ 0.05), whereas groups without shared letters are significantly different (P < 0.05).

**Figure S8. Autophagy activation and spermidine supplementation attenuate allergic pathology.** A mRNA expression of autophagy-related genes in jejunal tissue (n = 3). **B**,**C** Representative immunohistochemistry images and quantification of LC3-II expression (n = 5). **D** Body weight changes throughout the experimental period (n = 10). **E** Rectal temperature changes after final challenge in mice with or without SPD supplementation (n = 10). **F** Serum peanut-specific IgG1 and IgG2a levels (n = 8). **G** Representative photomicrographs of H&E- and toluidine blue-stained jejunal sections. Scale bar, 100 µm. Data are presented as mean ± s.e.m. *P < 0.05, ** P < 0.01, *** P < 0.001, **** P < 0.0001.

**Figure S9. Co-expression network analysis of SAT1 and long noncoding RNAs.**

**Figure S10. Cdk19os knockdown reduces systemic allergic responses.** **A** Body weight changes throughout the experimental period (n = 10). **B** Serum IFN-γ concentration (n = 10). Data are presented as mean ± s.e.m. *P < 0.05.

**Table S1 Primers for qPCR**

| Genes | Forward Primer（5’-3’） | Reverse Primer（5’-3’） |
| --- | --- | --- |
| *Odc1* | TCCTTGATGAAGGCTTTACTGC | ACATAGAACGCATCCTTATCGTC |
| *Srm* | AAAACCCGAGCACCAACTTC | TATGTCATTGAGGGCCTTCCG |
| *Sms* | GACCTTCAGAGTTACGACAGTG | GAACTATGGGTGGTAATCGCTTC |
| *Amd1* | TGTGGTTGTTGGTTGTTCGC | AACCAGACCTCCAGCAGTTTC |
| *Smox* | ACGTGATTGTGACCGTTTCG | GGGCTCCTCAAATTCAAGGAAG |
| *mSat1* | AAAATGGCAGCAGAGGAGTG | AAGCTTTGGAATGGGTGCTC |
| *rSat1* | GTTGCTATGAAGTGTCGCTGC | CACTGGACAGATCCGAAGCA |
| *mActin* | GAAGATCAAGATCATTGCTCC | TACTCCTGCTTGCTGATCCA |
| *rActin* | TTGCTATGAAGTGTCGCTGC | GGACAGATCCGAAGCACCTC |
| *mAtg5* | AAATGCAATGCAGCCGAAGC | AAGGTTCCACCATGGTCACC |
| *mAtg7* | ACACACACTGTTGCTGAAGG | TTGGTGTGCCACTTGAACAC |
| *rGpx4* | CCGTCTGAGCCGCTTATTGA | CTGCGAATTCGTGCATGGAG |
| *mMtor* | GCCTGATGGGATTTGGG | GGAGGTACTGGGTATCTGTGAA |
| *rAtg5* | AAGATGTGCTTCGAGACGTG | AGCTGACTCTTGGCAAAAGC |
| *rAtg7* | ATTGTCCTAAGGCAGTTGGC | ATGACTCAGCCAGCCTTTTG |
| *rMtor* | CTTCGGAACCTCCTCCC | CCCTCATTTCGGTCAGC |
| *mRAB* | CCGAGTCGCATGTCTCTGAT | AAACGTGATTCCCCAGAGGC |
| *mCdk* | CCTTGTTTTGGAACCTGGTGG | GGTAGCACAACTCACGAGCC |
| *mHox* | GCAGCTCTCCTTTGCTCAGCTACA | CTCTCTTGCCGCCCTCA |
| *mRab* | TCCCCAGAATGACCACAACG | TGACGCTCATTGTTGAGGCT |

**Table S2 Target gene primer sequence**

| Number | Sequence | GC content | Tm | Position |
| --- | --- | --- | --- | --- |
| lnc-probe 1 | GAGGAGGTAGCACAACTCACGAGC | 58% | 62℃ | 388-411 |
| lnc-probe 2 | CGCTGCAACTTTGCGCTACAGTTT | 50% | 63℃ | 531-554 |
| lnc-probe 3 | CCGGAATGAGCTCTAGAGCAAGGC | 58% | 63℃ | 668-691 |
| lnc-probe 4 | CCAAAGCCGGAGATAACCAGGCAC | 58% | 63℃ | 826-849 |
| lnc-probe 5 | AAAGATGATTGGCTGTGGGCCGTA | 50% | 62℃ | 995-1018 |
| lnc-probe 6 | CCCCTAGGTGAGCCTCGCCATTAC | 63% | 64℃ | 1174-1197 |
| lnc-probe 7 | AGTGGAAGAGAGCTGAAACGCGCG | 58% | 66℃ | 1366-1389 |
| Sat1-probe 1 | GCCAGTTCCTTGATCAGTCGCAGG | 58% | 63℃ | 51-74 |
| Sat1-probe 2 | ACATGGCGAACCCAACAATGCTAT | 46% | 62℃ | 206-229 |
| Sat1-probe 3 | CTCTTCACTGGACAGATCCGAAGC | 54% | 60℃ | 454-477 |
